# An interdisciplinary framework for derivation of occupational exposure limits

**DOI:** 10.3389/fpubh.2022.1038305

**Published:** 2022-11-30

**Authors:** Laura L. Maurer, Melannie S. Alexander, Ammie N. Bachman, Fabian A. Grimm, R. Jeff Lewis, Colin M. North, Nancy C. Wojcik, Katy O. Goyak

**Affiliations:** ExxonMobil Biomedical Sciences, Inc., Annandale, NJ, United States

**Keywords:** risk assessment, problem formulation, literature review, weight of evidence (WOE), point of departure (POD), assessment factors (AFs)

## Abstract

Protecting the health and safety of workers in industrial operations is a top priority. One of the resources used in industry to ensure worker safety is the occupational exposure limit (OEL). OELs are derived from the assessment and interpretation of empirical data from animal and/or human studies. There are various guidelines for the derivation and implementation of OELs globally, with a range of stakeholders (including regulatory bodies, governmental agencies, expert groups and others). The purpose of this manuscript is to supplement existing guidance with learnings from a multidisciplinary team approach within an industry setting. The framework we present is similar in construct to other risk assessment frameworks and includes: (1) problem formulation, (2) literature review, (3) weight of evidence considerations, (4) point of departure selection/derivation, (5) application of assessment factors, and the final step, (6) derivation of the OEL. Within each step are descriptions and examples to consider when incorporating data from various disciplines such as toxicology, epidemiology, and exposure science. This manuscript describes a technical framework by which available data relevant for occupational exposures is compiled, analyzed, and utilized to inform safety threshold derivation applicable to OELs.

## Introduction

Maintaining safe operations and protecting worker health is a clear priority in industrial settings. For select chemicals and industrial processes, OELs have been established by multiple stakeholders, including (but not limited to) regulatory bodies, governmental agencies, and expert groups and may apply on a global scale. Most notable are the American Conference of Government Industrial Hygienists (ACGIH^®^) Threshold Limit Values (TLVs), the Occupational Alliance for Risk Assessment (OARS) Workplace Environmental Exposure Levels (WEELs), and other national and regional OEL regulatory bodies.

Local regulatory limits should be the primary source for occupational exposure limits. However, some published OELs may lack the inclusion of the most recent relevant data. Also, for some chemicals and industrial processes, OELs have not been established or published by these stakeholders. In these cases, industry may need to develop their own internal OEL. Given that there are complexities in developing an OEL, including data integration, analysis and interpretation, transparency of the scientific process is important.

As a petrochemical company, we use a multidisciplinary framework which incorporates expertise in toxicology, epidemiology, exposure science, and/or industrial hygiene. The process begins with a review of published values such as the ACGIH^®^ TLVs, OARS WEELS, and national and regional OEL regulatory bodies, where applicable. Generally these values are adopted. An exception may be in cases where the scientific derivation of these published limits are not aligned with current scientific evidence; in this case, an internal OEL may be established. In the event that an OEL does not exist or is not supported by current science, we maintain a formal procedure for setting OELs that augment advisory and regulatory health limits to protect worker health. Where the science supports a more stringent limit, we adhere to the more stringent limit.

OEL reviews and development are triggered by several scenarios: (1) new products or manufacturing processes, (2) ACGIH Notice of Intended Change (NIC) to an existing TLV [Time Weighted Average (TWA) and/or Short-Term Exposure Limit (STEL)], (3) new or evolving science that suggests potential occupational health impacts, (4) business line, worker, or customer concerns or (5) periodic scheduled reviews of existing OELs. OEL review and development begins with assembling a multidisciplinary technical work team, followed by data assimilation and technical expert analysis where scientific expertise and principles of risk assessment are brought to bear.

**Figure 1:**
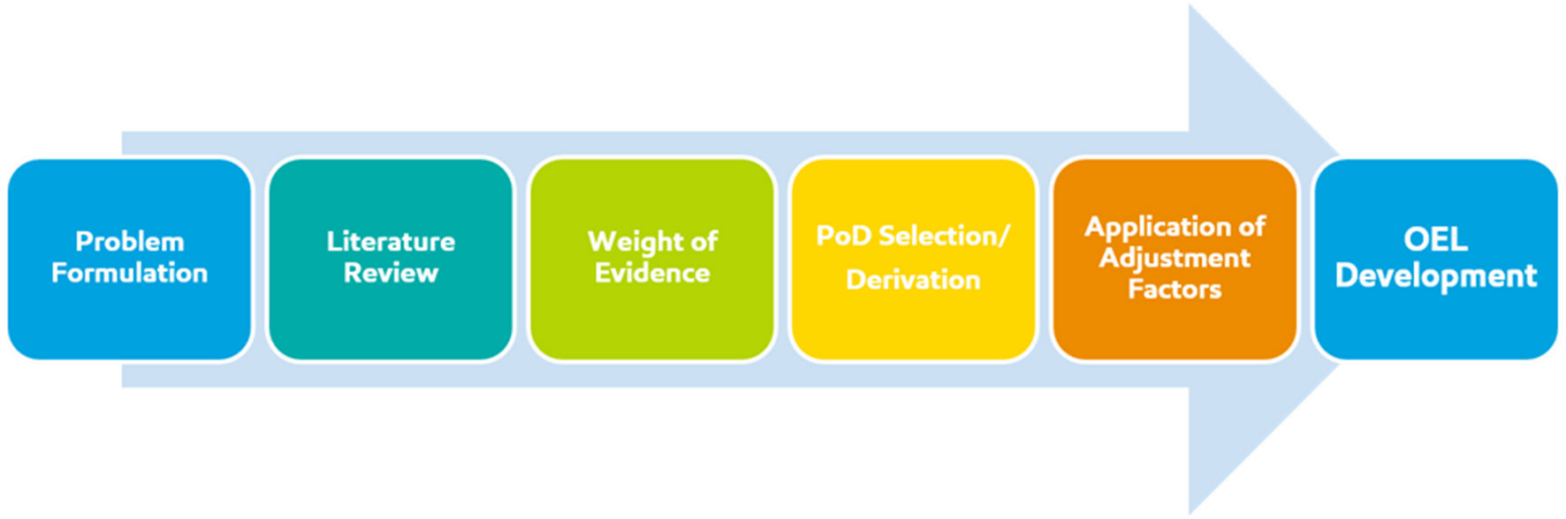
Overview of basic risk assessment steps involved in OEL derivation.

A special issue on the state of the science of OEL development was published in 2015 in the Journal of Occupational and Environmental Hygiene, which put forward contemporary advances in methodology and analysis of data relevant to OEL development, as well as a call for the use and implementation of advanced methods for OEL development [for e.g., (1–3)]. Advances in evaluation methods and emerging technologies continue to be published in this area [for e.g., (4, 5)]. The purpose of this manuscript is to share our learnings from this multidisciplinary approach to the collective OEL derivation process, starting with problem formulation and ending with uncertainty analysis. The technical assessment that is foundational to the development of a scientifically-derived OEL follows a sequence of steps which align with risk assessment frameworks (Figure 1). In this manuscript we discuss the technical attributes of each step: (1) problem formulation (define the scope of the question), (2) literature review (curate, sort, and evaluate all relevant data), (3) weight of evidence considerations (identify and gauge relative impact of key studies), (4) point of departure (PoD) selection/derivation (select the most sensitive adverse effect for hazard identification), (5) application of assessment factors (appropriately identify and quantify uncertainty related to PoD/key study), technical considerations (data quality, database uncertainty, integration of epidemiological and toxicological data), and the practical applicability of available information in the context of occupational settings.

We recognize that different organizations/industries may apply a range of inputs/problem formulations and scope to specifically address their needs. Regardless of these inputs, clear and sufficiently detailed documentation of decisions and rationale are central to transparency and reproducibility of the OEL process. Outside the scope of this manuscript is the comparison of approaches to OEL derivation globally; this has recently been undertaken by the OECD and the report is publicly available (6) and this type of comparison have been recently published, for example, Schneider et al. (4).

## Problem formulation for OEL development

Problem formulation is a critical first step in conducting any human health risk assessment (7–11). Problem formulation addresses the fundamental questions of “what do you need to know?” and/or “what decision do you need to make?” (10). First developed for ecological risk assessment (12), the problem formulation step establishes purpose, scope, and plan for collecting and evaluating information to guide effective use of resources at each stage of the assessment process and guards against collecting data with no clear sense of how they will be used. Additionally, by first focusing on describing and evaluating the specific problem to be solved, there is less tendency to immediately jump to all possible solutions, many of which may be inappropriate for the decision at hand.

Specific considerations to guide problem formulation have been tabulated (7) (Table 1). A more general framework to guide problem formulation (11), applicable to a wide range of assessment scenarios, can also be utilized. Explicit definition of these considerations promotes a flexible approach that allows a fit-for-purpose application of risk assessment methods. For example, comprehensive literature reviews on toxicity may not be necessary when the salient health effects are well-recognized, as is the case with benzene and hematological effects (however, as a best practice, periodic evaluations of the literature to identify new potential health hazards, as well as monitor advances in characterizing the dose response curve should be employed). As such, the scope of the problem can be refined when the health effects are well understood.

**Table 1:** Problem formulation considerations^a^.

**Element**	**Description**	**OEL considerations**
Scenario	Describes the occurrence and/or use of a chemical, biological, or physical agent	• Physical form of the substance • Monitoring method availability, limit of detection, and selectivity
Existing knowledge	Assembly and evaluation of all relevant information (chemical, physical and biological), including knowledge of chemical class and hypothesized modes of action	• All human and animal data on the substance • Alternative sources of data (e.g., read across, *in vitro, in silico*)
Context	Describes the conditions under which exposure may occur	• Operations (tasks and processes) associated with the substance(s) or chemical(s) • Co-exposures are generally out of scope^b^
	Describes the population to whom exposure may be associated	Individuals/populations who would be exposed, and exposure routes (e.g., inhalation, dermal) associated with the defined tasks and processes
Statement of the purpose of the assessment (e.g., priority setting, evaluation of a new use of an existing product, assessment of combined exposures)	• Determine decision point [e.g., target margin of exposure (MOE)] • Review available regulatory options (if applicable)	• Set an inhalation exposure limit that is measurable and health protective for most workers over a working lifetime (i.e., 40 years; adults ages 18–70; 8–12 h/day, 5 days/week) • Assess need for a STEL • Assess potential for skin sensitization

a As adapted from Embry et al. (7).

b An example of an exception to the consideration of co-exposures is the reciprocal calculation approach used to set OELs for hydrocarbon solvents, where “group guidance values” are assigned to similar constituents due to the similar toxicological properties and additive effects demonstrated in toxicological studies (13).

The primary purpose of the problem formulation step is to adequately define what is in scope and what is out of scope to ensure appropriate resources and expertise are engaged to solve the defined problem. In the context of setting OELs, a problem formulation statement would include relevant information on the scope of the OEL, such as new products or manufacturing processes or new or evolving science that suggests potential occupational health impacts. The OEL process aims to set an inhalation exposure limit that is measurable and health protective for most workers over a working lifetime (i.e., 40 years; adults ages 18–70; 8–12 h/day, 5 days/week), while also assessing the need for a STEL, importance of dermal routes of exposure, and skin sensitization concerns.

OELs are frequently communicated as 8 h TWA, 15 mins STEL, or both. TWA typically applies where there is a health effect from repeated exposures to a relatively continuous exposure concentration (i.e., not solely peak or intermittently high exposures). The TWA is more frequently associated with observed effects following repeated exposures, where effects are thought to be primarily time- and concentration-driven (as opposed to solely concentration-dependent). STEL typically applies where there is a health effect resulting from a single exposure or peak exposures may result in effects not observed following relatively continuous exposure concentrations. The STEL is more frequently associated with effects such as respiratory irritation, where effects are thought to be primarily concentration-driven (as opposed to both time- and concentration-dependent) or dose rate-dependent toxic effects (i.e., narcosis of sufficient degree to increase the likelihood of accidental injury, impaired self-rescue, or reduced work efficiency). Thus, the problem formulation step includes consideration of the nature of the health effects and if those effects justify a TWA and/or STEL. It may be important to recognize that even if the key effect justifies only a TWA, a secondary effect may justify a STEL recommendation. For example, if liver injury is the key effect and a TWA is derived, but an exposure only marginally higher would result in respiratory irritation, a STEL might also be recommended.

Recommendation for a STEL only (no TWA recommended) may be considered when available information supports potential solely for acute effects and repeat exposure effects are secondary to the acute effect. Respiratory irritants can be an example of this scenario. If a chemical's mode of action for repeat exposure effects is dependent on repeated irritation to the lung, but a STEL will prevent lung irritation, then the STEL could be appropriate to consider for the OEL. Where there is a TWA only (no STEL), an excursion limit, similar to the ACGIH Peak Exposures guidance of three times the TWA, is recommended to limit short-term high exposures.

Another factor to consider during problem formulation is the nature of potential exposure in the workplace to ensure that the assumptions used to derive the OEL align with the exposure scenarios of interest. Such consideration may include characterization of the exposed population (i.e., worker groups), as well as the work environment (e.g., operating conditions) and tasks performed, which inform the source and form of the substance in the workplace and the primary route(s) of exposure. If the exposures in the workplace are sufficiently different from that of the science behind the derived limit, the OEL might not be relevant (e.g., ACGIH TLV for chromium; see discussion for details) and may lead to inappropriate risk management decisions.

## Literature review

**Figure 2:**
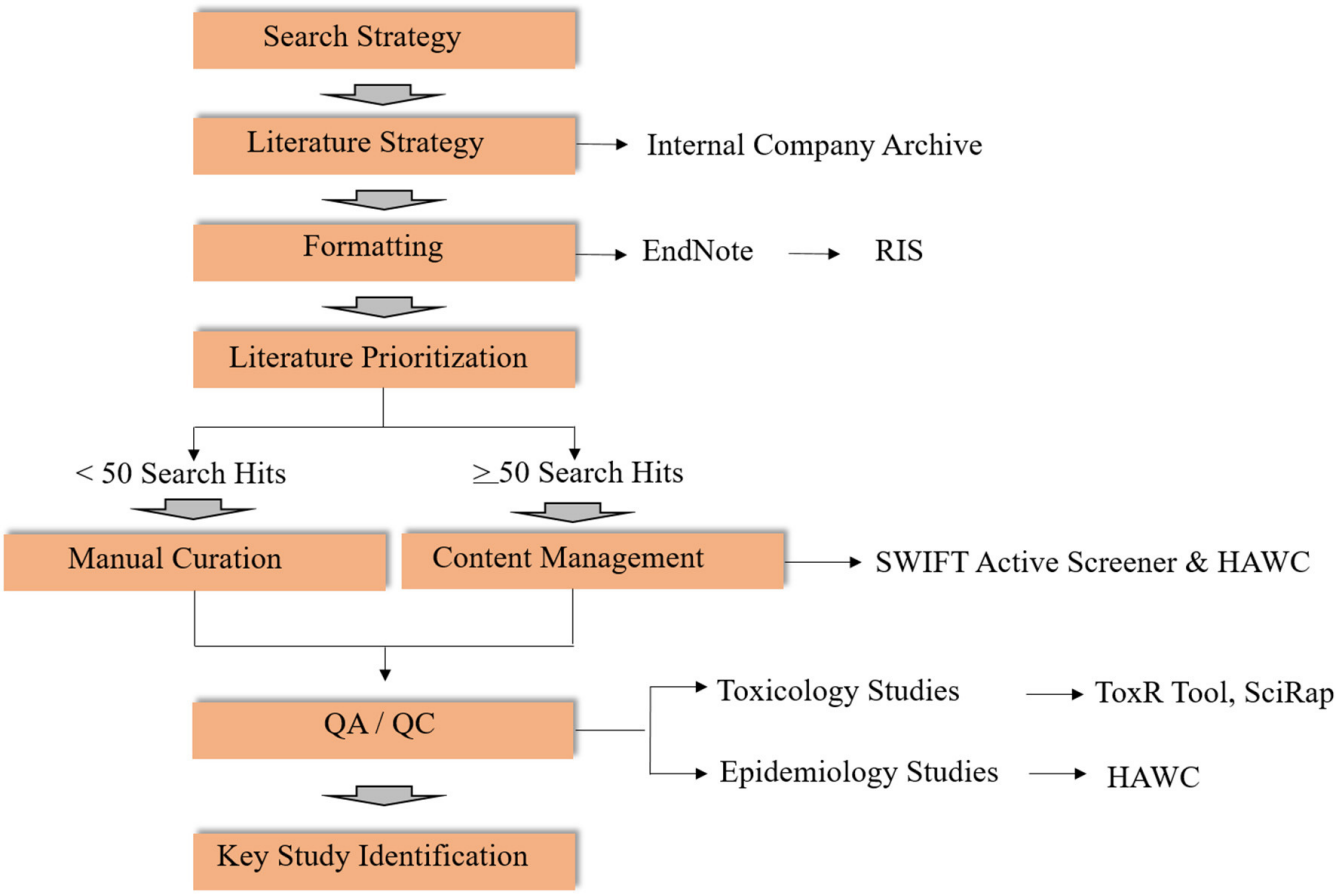
Workflow for literature review for activities related to OELs (RIS, Research Information Systems; SWIFT, Sciome Workbench for Interactive computer-Facilitated Text-mining; HAWC, Health Assessment Workspace Collaborative; SciRap, Science in Risk Assessment and Policy; QA/QC, Quality Assessment/Quality Control).

Literature reviews and literature-based data synthesis is the second key step in OEL development (Figure 2). Though some of the elements of a systematic review (14, 15) are used to identify and evaluate potentially relevant studies in this context, the literature review in developing new and reviewing existing OELs is considered broader in scope. This is because a clearly specified research objective, which is usually defined in a Population-Exposure-Comparator-Outcome (PECO) statement, is not typically included. Here we outline the methods for conducting a literature review and synthesis for two OEL development scenarios (Figure 2). Software-assisted approaches for large bodies of literature are highly recommended to improve efficiency in time, resources and documentation. Elements of the workflow can also be adapted to be fit-for-purpose, and depends on the body of literature at hand.

In terms of search strategy, literature searches for OEL derivation may be conducted in the context of (1) *de novo* OEL development or (2) periodic scheduled review cycles. For *de novo* OELs, a search strategy is developed by a multidisciplinary team, ideally in collaboration with an information specialist. For the periodic reviews, previous OEL documentation can inform search terms, together with review and modification of the search strategy if appropriate. Once a search strategy has been established, an information specialist conducts the literature search in appropriate databases (e.g., PubMed, ProQuest, internal company archives). If multiple databases are used, duplicate entries should be excluded using reference management software, such as Endnote. Once duplicate references have been removed, the EndNote library can be exported as a Research Information Systems file (.ris). RIS file formats can be imported to various bibliographic software and are compatible with other text-mining tools, including SWIFT Active Screener (SWIFT is an acronym for “Sciome Workbench for Interactive computer-Facilitated Text-mining”) (16), and Health Assessment Workspace Collaborative (HAWC) (17, 18).

Because manual curation for a large number of search returns is labor- and resource-intensive, content management using software tools in combination with subject matter expert screening is strongly recommended. As an example, two web-based, collaborative software tools may be useful: SWIFT-Active Screener and HAWC.

1. SWIFT-Active Screener (16): SWIFT-Active Screener (https://www.sciome.com/swift-activescreener/) is a commercial web-based platform designed to facilitate literature prioritization for unscreened articles based on screened articles that were included or excluded using an underlying statistical model. The .ris file exported from EndNote can be imported into Active Screener. After screening, results can be exported in standard data formats compatible with another content management tool, HAWC.
2. HAWC (17, 18): HAWC (https://hawcproject.org) is a freely-available, web-application and content management tool designed to support the systematic review process, including search hit categorization, content extraction, risk of bias analysis, and data visualization. HAWC therefore provides a convenient platform used to capture key study data.

For the development of new OELs in particular, it is recommended to start with compendium documents (e.g., ACGIH and/or NIC documentation, SCOEL, systematic reviews, etc.) to facilitate rapid identification of the highest quality studies, regardless of the software tools being used to organize the results of the literature search. Although compendium and other summary documents will help to expedite the literature review process by narrowing scope and clarifying the most sensitive health endpoints associated with a compound, review of the original paper(s) referenced in the compendium document(s) is still essential. Outdated compendium documents should be utilized with caution and underscore the importance of evaluating the most relevant and informative studies identified in the literature search.

## Weight of evidence

After the relevant literature has been identified, the next step in the hazard assessment and OEL derivation process is synthesis of the available lines of evidence (LOE), which often include diverse and not readily comparable types of data (e.g., animal studies, epidemiological studies, *in vitro* mechanistic studies, physical-chemical properties) in order to make a single, health-protective decision. The integration and critical weighting of all suitable, available studies using predefined, scientifically justified criteria for both quality and relevance to the problem formulation is known as a weight of evidence (WOE) assessment. Several regulatory agencies have recently published frameworks or perspectives on approaches to integrate and weight different LOE in hazard identification, including EFSA, Health Canada, and the National Toxicology Program (19–21). Although each organization has slight nuances, each includes the following three steps: establishing the LOE (including selection of relevant studies and assessing the quality of the studies), assessing confidence in the LOE, and integrating the LOE to express a single WOE hazard conclusion. The following sections highlight key considerations for each of these processes.

### Establishing LOE

A critical part of establishing the LOE is a clear and transparent process to select individual studies to make up the body of evidence. Without clear criteria, a WOE assessment tends to rely on expert judgement, resulting in variable conclusions with little insight into the underlying reasons for the variability. For the purposes of setting OELs, inclusion criteria may be defined as direct assessment of a hazard endpoint (e.g., acute toxicity, irritation, sensitization, genetic toxicity, carcinogenicity, reproductive or developmental toxicity) in either animals or human subjects or assessment of mechanistic information. Such mechanistic studies may identify previously unknown adverse effects or change previous conclusions on relationships between exposure and effect levels. Additionally, mechanistic data can inform as to the human relevance of findings observed in animals (22).

For the studies considered relevant (i.e., meet the inclusion criteria described above), a quality assessment may be conducted to determine the impact of study design on the validity of the link between effect and exposure, following the National Toxicology Program's (NTP) Office of Health Assessment and Translation's (OHAT) Risk of Bias tool (23). The intent of this step is to identify limitations that could potentially introduce a systematic bias that would threaten the validity of the study's findings. The Risk of Bias tool asks a series of questions to address various types of bias (selection, confounding, performance, attrition/exclusion, detection, and selective reporting), with different considerations per study type (human controlled trial, cohort, case-control, cross-sectional, and case series/case report, experimental animal studies). As an example, confounding bias is the major threat to an observational study's validity, as occupational epidemiology studies often do not adjust for co-exposures and lifestyle issues such as smoking (24).

### Assessing confidence in LOE

The overall confidence in the body of evidence provides an indication of the likelihood that the available study findings provide an accurate representation of the association between exposure and effect. Characterizing confidence in the evidence takes into account both the amount of data available and professional judgement on the consistency, relevance of study design to directly and/or precisely measure the effect, etc. It is recognized that this step in the process requires scientific judgment; however, a transparent, systematic process to include all relevant data and to document the rationale for exclusion and confidence decisions provides a foundation for further discussion as needed. As noted above, organizations may use varied processes to assess confidence; the critical element is that the process followed is clearly communicated. Table 2 provides an example of how confidence decisions may be documented in a systematic manner.

**Table 2:** A practical example to an approach to the systematic and transparent documentation of a WOE assessment^a^.

**Step 1. Establish the LOE, including quality assessment of the individual studies per LOE**.			
**Step 2: Assign confidence rating to each hazard endpoint per LOE**			
**LOE**	**Considerations informing confidence** ^b^	**Confidence description**	**Confidence rating**
Hazard endpoint 1/Animal data	• Clear dose response • Large magnitude of effect/meets UN GHS classification criteria^c^ • Consistency across disparate study designs • Mode of action considerations	High confidence that additional studies and/or data are unlikely to change the understanding of the exposure/effect relationship	High
Hazard endpoint 2/Animal data Hazard endpoint 3/Animal data, Etc.	• Lack of dose-responsiveness • Small magnitude of effect • Indirect measurement of effect • Inconsistent findings across animal models/species/study designs	Low confidence in accurate representation of the exposure/effect relationship; new data likely to change the representation	Low
	No studies identified	No studies identified	No data
Hazard endpoint 1/Epidemiological data	• Quantitative/measured exposure data • Clearly described exposure history, including shape of the exposure distribution • Repeated air sampling • Accounts for co-exposures and/or confounders • Study population sizes with substantial effect observations (e.g., >5 cases) • Diverse study populations or meta-analyses	High confidence that additional studies and/or data are unlikely to change the understanding of the exposure/effect relationship	High
Hazard endpoint 2/Epidemiological data Hazard endpoint 3/Epidemiological data, etc.	• Case reports, accidents, intentional misuse, etc. • Qualitative exposure metrics • General population studies • Exposure to other stressors (e.g., excessive smoking, alcohol/drug use) • Small or non-diverse study populations	Low confidence in accurate representation of the exposure/effect relationship; new data likely to change the representation	Low
	No studies identified	No studies identified	No data
**Step 3**. Translate the confidence ratings into the level of evidence			
**Effects observed?**	**Confidence rating**		**Level of evidence for effect**
Yes	High		High potential
Yes	Low		Moderate potential
No	High		Low potential
No	Low		Low potential
No data	Low		Note: in an absence of data, adjustment factors for database quality should reflect the increased uncertainty or potential underestimation of effect

a Process adapted from Rooney et al. (21).

b Considerations adapted from Rooney et al. (21) and (25). For more detail on epidemiological considerations that may influence confidence, (see Appendix).

c United Nations (UN) Globally Harmonized System of Classification and Labelling of Chemicals (GHS): Eighth Revised Edition (2019). https://unece.org/ghs-rev8-2019.

### Integrating LOE

Different approaches can be taken to integrate effects information obtained in separate LOE. Most commonly, all effects (including animal effects and human health effects) are shown together in tabular format, and endpoints with an effect deemed to have a high level of evidence (i.e., high potential) are considered as potential points of departure for OEL derivation.

Ideally, a biological-pathway approach should be considered to integrate both the animal and human LOE, as well as to put mechanistic information into the context of the apical outcomes derived from observational animal and human studies. In this approach, the effects observed at a molecular or tissue level, obtained in *in vitro* or animal studies, are linked to apical outcomes, often observed in animal or epidemiological studies. In this way, observations across distinct LOE can be assessed for both dose- and temporal-concordance and consistency across species. For example, an agent characterized as being particularly toxic to a specified organ system *via* toxicology studies paired with unadjusted epidemiologic results might suggest that, whatever level of confounding might reasonably exist, the epidemiologic findings are reasonably valid.

**Figure 3:**
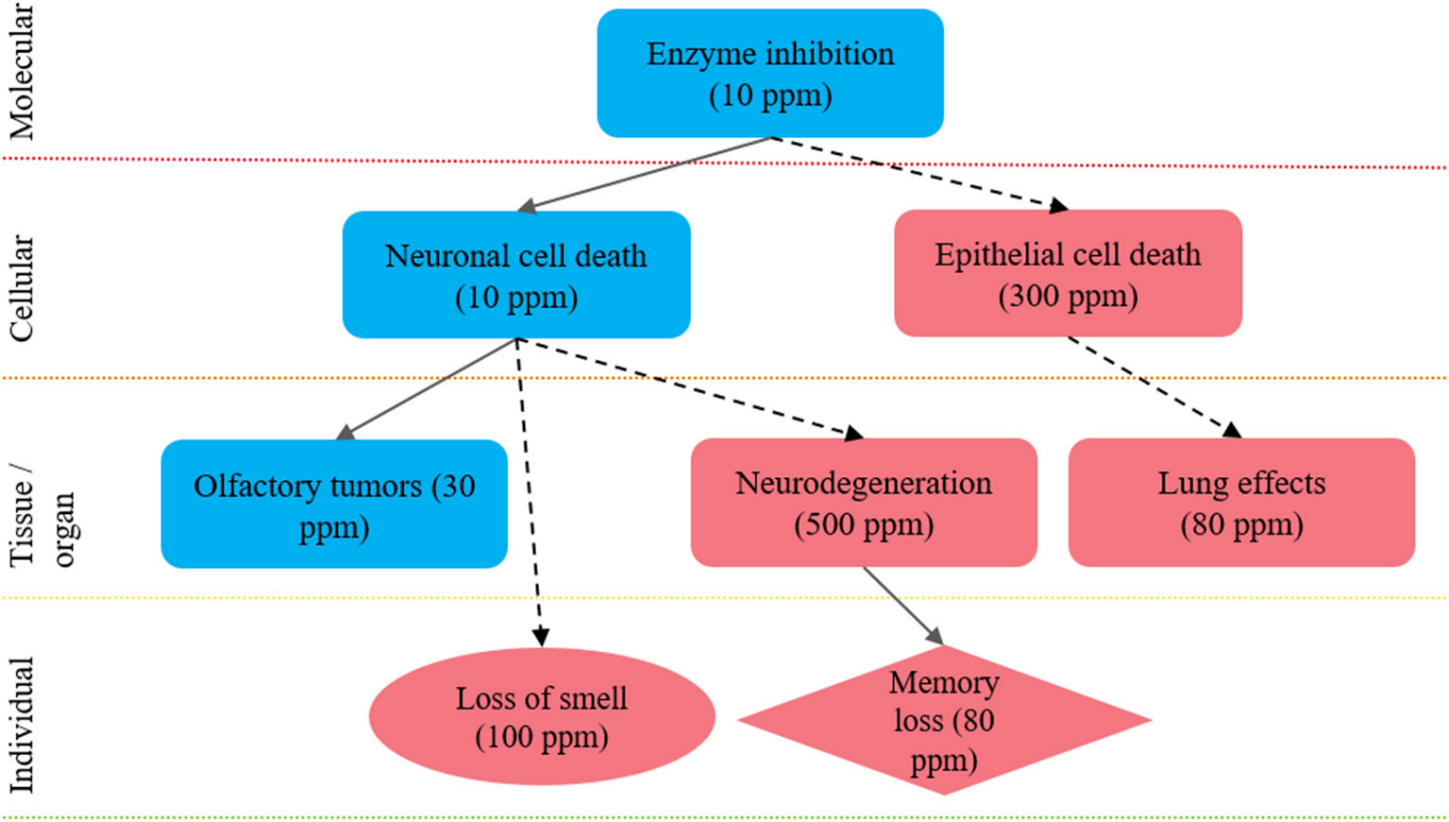
Integration of health effects into a biological pathway network to inform future selection of a point of departure: example biological pathway network approach to integrate health effect data derived from distinct LOEs to inform selection of a health-protective POD. The key events shown here are proposed to lead to hallmark effects associated with hydrogen sulfide exposure: nasal tissue outcomes; neurological tissue outcomes and pulmonary tissue outcomes. The shape of each key event indicates the LOE: rectangle, animal data; circle, epidemiological data; and diamond, both animal and epidemiological data. The level of evidence supporting linkages between key events is shown by the arrows: solid arrows, quantitative evidence; dotted arrows, qualitative evidence. The color of each shape reflects a relative distinction between high- and low-dose effects, where effects observed at <30 ppm are considered low dose effects (blue) and effects observed at >30 ppm are considered high-dose effects (red). Figure adapted from Goyak and Lewis (26).

To demonstrate the organization of effects into biological pathways, (see Figure 3), which summarizes effects observed after exposure to hydrogen sulfide in mechanistic studies (e.g., enzyme inhibition), animal studies (e.g., nasal lesions, lung effects, memory impairment), and epidemiological studies and/or human case reports (e.g., loss of sense of smell, memory impairment). See Goyak and Lewis (26) for more detailed discussion of this example biological pathway network. Integration of the effects data obtained from different LOEs can increase the overall confidence in the body of evidence. For example, through demonstration of consistency in effect across disparate study designs, by highlighting the distinction between low- and high-dose effects, and by showing dose- and/or temporal-concordance across the entire pathway.

Regardless of the method, the overall goal of integrating the LOE is to characterize the evidence base and assess confidence in each possible outcome, in order to inform subsequent steps in the OEL derivation process. Specifically, the confidence descriptors are used to inform both PoD selection (e.g., an endpoint with low confidence is likely not an appropriate candidate for the point of departure) and application of assessment factors (e.g., an endpoint with no supporting data indicates low confidence and usage of additional assessment factors may be considered).

## Point of departure selection

A point of departure (PoD) refers to a dose (either measured empirically or modeled using dose-response data) at which an adverse effect occurs as a result of a specific exposure. The International Programme on Chemical Safety's (IPCS) definition of adversity is helpful in PoD selection (27):

“*Change in the morphology, physiology, growth, development, reproduction, or life span of an organism, system, or (sub) population that results in an impairment of functional capacity, an impairment of the capacity to compensate for additional stress, or an increase in susceptibility to other influences.”*

Utilizing a PoD which reflects an accurate and holistic scenario for occupational exposures is a critical aspect of an OEL determination. This section details approaches to PoD selection which consider unique aspects of human and animal datasets, as well as scientific criteria which aid in the selection of a PoD relevant for an occupational exposure to that substance. Because considerations for PoD selection can vary based on study design, underlying assumptions, extrapolation potential, the human and animal considerations are separated in this section. However, it is best practice to consider all available data together in a WOE approach to select the most appropriate study for the PoD.

### PoD selection based on human data

If an adverse health effect is identified, a PoD can be selected. In cases where a reported human health effect(s) is unsuitable for determining an OEL, the available animal toxicity data to select the PoD should be considered. If there are no available animal toxicity data for the substance, read across data is in scope to select a PoD.

It may be challenging to identify a PoD or threshold of effect from human data, because in many cases the study was not designed to allow the dose-response relationship to be characterized quantitatively or a threshold of effect to be identified. A dose/concentration level which corresponds to a no or low effect level is selected as the PoD, the starting point for low dose extrapolations (28). The PoD can be the no-observed-adverse-effect level (NOAEL), the lowest-observed-adverse effect level (LOAEL), or derived using dose/concentration-response modeling, e.g., the benchmark dose (BMD).

In selecting the PoD from human data, consider the following features of the PoD regarding irritation as an endpoint. For human studies, with only subjective symptoms, such as irritation, reported for local effects, consider selecting the concentration associated with clear to moderate irritation as the PoD (since very slight to slight discomfort subjective irritation is often reported at near zero exposure) (29). If human data are limited to chemosensory irritation (trigeminal nerve stimulation, reported as burning, stinging, headache, discomfort), the assessor may consider using animal Alarie data to support the human-derived PoD because the Alarie data provides an objective measure of irritation. Alarie data refers to the historical use of an animal bioassay to predict sensory irritants in humans (30). The correlation drawn from this animal bioassay still has practical application to OELs in this context, to support conclusions on human data when the human data is of lower quality or potentially ambiguous interpretation.

Categorical exposure assessment is frequently used in environmental or occupational epidemiology studies. The descriptive statistics for exposure categories (mean, median, upper or lower limits for range in exposure categories) are potential quantitative inputs for PoDs in human studies. The central tendency of individual exposure categories may be preferred if the category interval is not large, but if the interval between the upper and lower bound of a category is large it may be preferable to adopt the upper or lower bound of the interval as the PoD, depending on whether the exposure category would be considered a NOAEL or LOAEL. Consideration of the quality of the exposure assessment method may also be appropriate in informing the scientific confidence in exposure categories.

In some cases a regression model may be available for predicting the endpoint of interest. It may be possible to use a regression model similarly to a BMD. In this scenario, the assessor may identify a specific effect size on the critical endpoint (i.e., the amount of risk to be used), then calculate the corresponding exposure concentration from the regression model to identify a PoD. The rationale for the selected effect size should be documented in the OEL. If this approach is considered, consultation with a statistician may be required to understand the underlying model constraints and resultant uncertainties that may be introduced into the PoD selection.

### Approaches to PoD selection from animal studies

**Figure 4:**
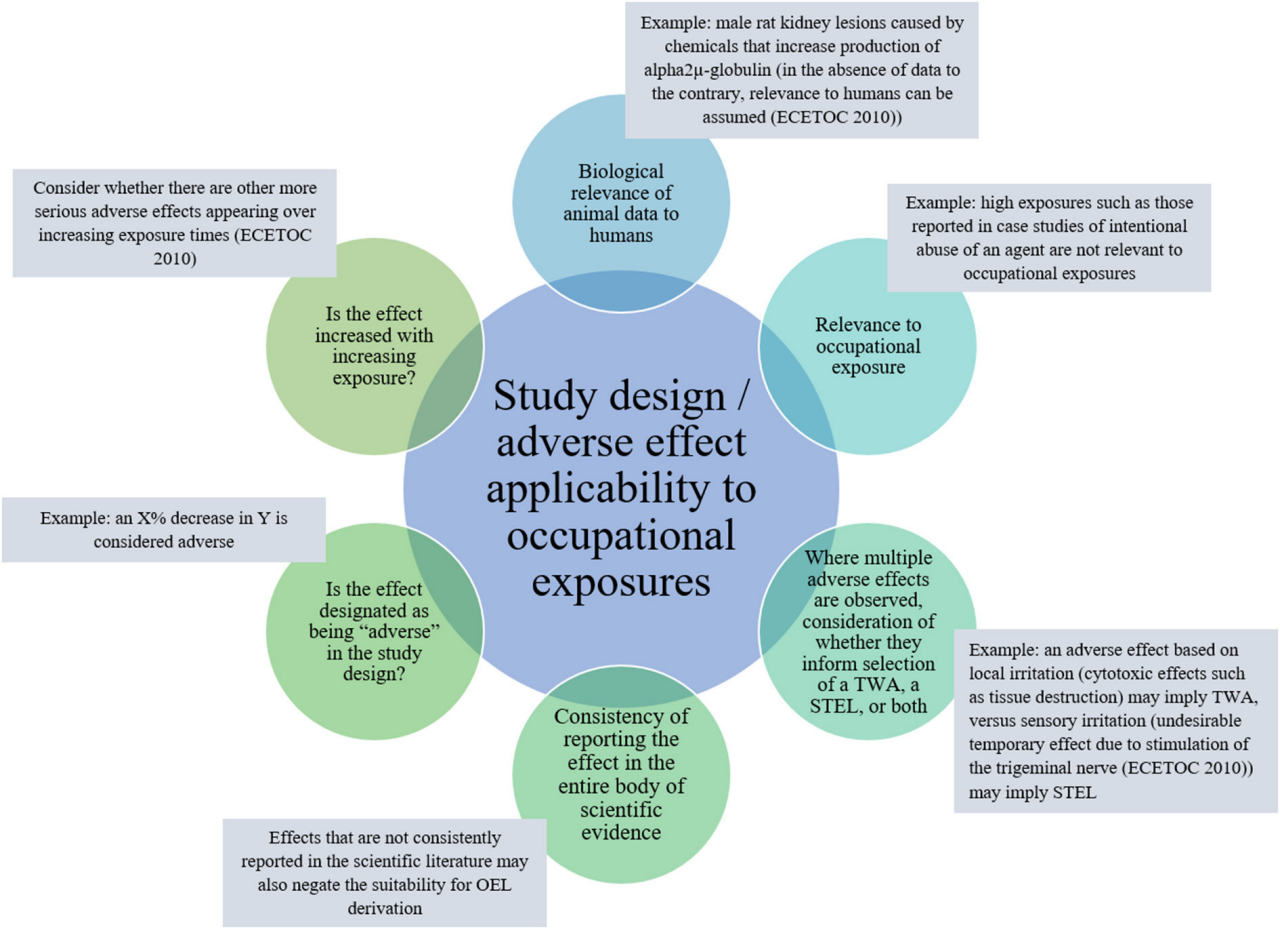
Key adverse health effect: effect and study design features to consider regarding applicability to OEL development.

Two approaches to PoD selection are common in OEL development from animal studies, NOAEL/LOAEL and BMD approaches. The approach selection is likely dependent on the available study design (for considerations on applicability of adverse effect and study design to OEL development, see Figure 4). Primary considerations useful in guiding selection of an approach are the number of dose groups, group sizes, dose spacing, and approximated dose-response inflection point.

#### NOAEL/LOAEL approach

A NOAEL/LOAEL approach has been traditionally applied in toxicology. It commonly relies on one or more pair-wise comparisons of a control group to exposed group(s). When an adverse effect is observed, the NOAEL is the **highest** dose where a statistically significant difference does **not** exist between the control and exposed groups. The LOAEL is the **lowest** dose where an adverse effect shows a statistically significant difference from the control group. In this context, both statistical and biological significance should be considered. Some expert judgment may need to be applied when statistical comparisons are borderline significant **or** when effects are statistically significant, but not biologically significant or relevant to humans when considering the animal model used in the study design. The NOAEL/LOAEL approach may be preferred if there are a limited number of experimental groups. A NOAEL/LOAEL approach is the only realistic approach if there are two dose groups, as there is insufficient information in such a design to permit dose response assessment.

Two primary weaknesses of a NOAEL/LOAEL approach is that it can become strongly dependent on the statistical power for comparisons between groups and the spacing of the dose groups. In a study design with low statistical power, the NOAEL/LOAEL approach may be prone to misestimating the true NOAEL/LOAEL because a true effect may not be observed as statistically significant (due to limited sample size or chance). A scenario in which one additional study subject would have changed a result to be statistically significant is distinctly different from needing to triple the group sizes. With sample sizes of five to ten animals per group, the influence of variability, random effects, and multiple comparisons may increase the chance that a true effect is not statistically significant. The spacing of dose groups can also be a weakness. Because the NOAEL/LOAEL approach requires the selected PoD to be one of the test concentrations, wide intervals between doses or tests performed well above the NOAEL can occur. Wide dose spacing may obscure the true threshold, leading to selection of a NOAEL that is far below the true PoD. In studies where adverse effects occur in all exposed groups, there can be substantial uncertainty about where the true PoD is.

When applying a NOAEL/LOAEL approach in OEL development, consideration of how statistical power may influence NOAEL/LOAEL determination should be deliberately assessed. Consideration of the historical control range for a specific lab and strain of animal model can be helpful in assessing results that are not statistically significant, but may be biologically significant. Consideration of dose spacing can also be a consideration in the assessment factor for LOAEL to NOAEL extrapolation, as wide dose spacing could introduce uncertainty in the true NOAEL.

#### BMD approach

The BMD approach addresses several weaknesses of NOAEL/LOAEL approach, but is not without its' own weaknesses. In BMD modeling, multiple statistical models are fit to the observed data in an effort to identify the model that best represents the observed data. The modeler identifies a Benchmark Response (BMR) that is consistent with a non-adverse effect, and the dose corresponding to that BMR is identified as the BMD. All the statistical models have some uncertainty with regard to the precise location of the true BMD, thus the 95th percentile lower confidence limit (BMDL) is generally selected as the PoD for a selected BMR (31).

Model selection when multiple appropriately fitting statistical models are available is one of the challenges of BMD analysis. In selection of a single statistical model the assessor may introduce a “model selection error.” US EPA (31) and EFSA Scientific Committee et al. (32) have articulated guidance on model selection, both of which consider model fits but compare by different measures. The risk of model selection error may be decreased by applying model averaging techniques (33). US EPA BMDS has integrated model averaging for some statistical models, and web-based tools for deriving a model average BMD are also available (34). In documenting the BMD analysis the rationale for selected model should be provided by the assessor. As an additional consideration, model averaging does not mean using individual BMD or BMDL estimates from different models to calculate a mean (sometimes called an average BMD or BMDL), but instead using whole dose response models with different mathematical weights to calculate a model average. A discussion of model averaging methods is beyond the scope of this summary information.

One additional element to keep in mind for the BMD approach, the BMD software offers the analyst a choice for risk type: added risk or extra risk. Both are different approaches to handling the background incidence of an effect. When the background incidence is zero there is no difference, but if the background incidence is high it can create a major difference in the calculated BMR. As background incidence increases, the calculated risk will increase linearly. The result of the higher calculated risk will be a lower BMD and BMDL. If background incidence of the response is high (80–90%) the calculated BMD and BMDL will differ substantially based on the selected risk type, with the Extra Risk value being lower. Because of the calculation method Extra Risk will always be equal or more conservative than Added Risk. When using BMD software for a quantal (dichotomous) endpoint measurement it is desirable to document values using both approaches to risk.

### Adjustments to PoD—inhalation exposure

If the key study used to identify a PoD is based on inhalation there may be additional considerations that cause an assessor to adjust the PoD because breathing rates and particle depositions can differ between laboratory animals and humans. The PoD value identified following adjustment based on respiratory differences has historically been called the “Human Equivalent Concentration” in some documentation. For further discussion and guidance on the Human Equivalent Concentration, consult the EPA Methods for Derivation of Inhalation Reference Concentrations and Application of Inhalation Dosimetry (35). The complexity of inhalation dosimetry can lead to some confusion with regards to the appropriateness of applying allometric scaling in route to route extrapolation (see next paragraph on allometric scaling). ECHA (36) provides a flow chart for extrapolating an oral exposure to an inhalation exposure for both the general public (Example R.8-1, p. 58) and for an occupational exposure (Example R.8-2, p. 59). These flow charts describe approaches to allometric scaling within the context of route-to-route extrapolation to inhalation, and under which circumstances allometric scaling should explicitly be performed, or whether it has been implicitly addressed in other aspects of the extrapolation procedure.

Briefly, an interspecies allometric scaling assessment factor is *not* applied if a PoD adjustment for inhalation is applied. Inhalation scales nearly allometrically, so adjusting the PoD based on intraspecies differences in inhalation replaces allometric scaling (i.e., do not adjust breathing rates *and* apply allometric scaling). Route-to-route extrapolations, where an oral exposure in rodents is extrapolated to an inhalation scenario, are likely to apply allometric scaling [see examples R.8-1 and R.8-2 (36)]. Where a rodent inhalation exposure is extrapolated to a human inhalation scenario, the breathing rates are more likely to be applied. Adjustment in breathing rate differences for resting animals compared to working humans can be included in the PoD adjustment. Because allometric scaling pertains to resting energy use, adjustment for the difference between resting and working breathing rates is appropriate even when allometric scaling has been applied. Most rodent inhalation studies are performed with animals at rest, resulting in a comparatively smaller volume of air consumed compared to that of a physically active worker. If the adjustment is performed in PoD adjustment the calculation, and source for breathing rate data, should be identified in the documentation.

For particle exposures (aerosol, dust, mist) the comparative deposition fraction can be calculated from common laboratory animal species and humans if particle size and distribution information are available. The comparative deposition fraction can be used to adjust anticipated dose. The Multiple-Path Particle Dosimetry model (https://www.ara.com/products/multiple-path-particle-dosimetry-model-mppd-v-304) can be used for calculation (37, 38). Assessors using Multiple-Path Particle Dosimetry model for PoD adjustment should document the parameters and source of the parameters used for modeling.

## Assessment factor (AF) application

The principles underpinning the selection of the PoD (e.g., study quality, route of exposure, animal or human study as key study, duration of exposure) characterize and inform uncertainties that need to be addressed in further steps to derive the OEL. These uncertainties are addressed by applying assessment factors, which introduce quantitative conservativism to the PoD. These AFs are based on physiological differences between human populations as well as animal models and humans, extrapolations for exposure route and duration, and the quality of the overall database on the substance.

This section introduces the application of appropriate AFs to a key study from which the PoD has been derived. Scientifically justifiable AF selection is a critical component of the OEL derivation process, as it accounts for the uncertainty around aspects of the key study. An aim of this section is to articulate assignment of appropriate ranges or values to use when assigning an AF, in addition to when uncertainties in the dataset may require additional expert judgement.

Human datasets and animal datasets are inherently different. There are two primary sources of human data from which an OEL may be derived: (1) observational studies and (2) experimental/intentional exposure. In general, observational studies are well suited for studying chronic, long-term endpoints, including cancer; studies often involve worker populations of sufficient size to validly estimate risk. Experimental human studies are generally conducted to examine a focused set of acute, transient heath endpoints. Sample sizes are often small, and study subjects are generally younger and healthier relative to the workforce.

When developing the rationale for AFs, there are five main areas to account for: (1) interspecies extrapolation, (2) intraspecies adjustment, (3) exposure duration of the study, (4) dose-response extrapolation, and (5) database quality. There are publicly available guidance documents which detail considerations for application of assessment factors (29, 36, 39). Each of these guidance documents utilizes scientific principles which often, but not always, agree on recommendations for appropriate AF selection and application. For a comprehensive table comparing the recommended ranges for each AF between ECHA and ECETOC, (see Table 1) in the ECETOC guidance (29).

### Route to route extrapolation

The route to route extrapolation factor accounts for uncertainties when the key study uses a route of exposure which is different from the exposure meant to be understood in the workplace. Where a route to route extrapolation is applied, the assessor should document their rationale for all factors used (even if the factor is 1, which necessitates the justification for why a chemical's disposition would not vary among exposure routes). As outlined in their guidance on deriving AFs for human health risk assessment (39), the consideration of the following factors for specific chemicals may lead the assessor to recommend an AF for route to route extrapolation:

As an illustration of route to route extrapolation, consider three examples^*^:

1. Extrapolation from a rat oral gavage study to an inhalation OEL, where available toxicokinetic information indicates oral absorption is 90%. The daily exposure at the PoD was 100 mg/kg/d. The nominal dose is adjusted for oral absorption to 90 mg/kg/d (100 mg/kg/d ^*^ 90% absorption = 90 mg/kg/d absorbed dose). No additional adjustment for route to route extrapolation is suggested.
2. Extrapolation from a rat dermal study to an inhalation OEL, where available toxicokinetic information indicates dermal absorption is 5%. The daily exposure at the PoD was 100 mg/kg/d. The nominal dose is adjusted for dermal absorption to 5 mg/kg/d (100 mg/kg/d ^*^ 5% absorption = 5 mg/kg/d absorbed dose). No additional adjustment for route to route extrapolation is suggested.
3. Extrapolation from a rat oral gavage study, where an acceptable toxicokinetic model (may be one, two, or many [PBPK] compartment) is available. The daily exposure at the PoD was 100 mg/kg/d, resulting in a model predicted time-weighted blood concentration (AUC_0 − 24*h*_ = 7,000 μg h/ml). Using the model, the same AUC_0 − 24*h*_ is achieved with a 30 mg/m^3^ for 8 h exposure, which is then utilized as the PoD. No further adjustment for route to route extrapolation is suggested.

^*^These examples do not take into account any chemical-specific knowledge on ability to extrapolate between exposures in air and exposures to the skin; assessor should consider these and other aspects of ADME dynamics which are chemical-specific when doing route-to-route extrapolations.

### Interspecies

The interspecies AF accounts for the extrapolation between the average study animal and the average human. This extrapolation is primarily based on differences in metabolism between the animal species utilized in the study and humans, and accounts for toxicokinetic and toxicodynamic differences. In the absence of substance or species-specific data, ECETOC guidance recommends using allometric scaling factors to inform the interspecies AF under certain conditions (39). Allometric scaling is defined as biological changes in an organism related to proportional changes in body size. In the context of the interspecies AF, the principle of allometric scaling is used to account for differences in basal metabolic rate between animals and humans. Most toxicokinetic differences can be explained by differences in the basal metabolic rates between species—this is based on the principle that metabolic rates of smaller animals are faster than that of humans. This difference means that humans “would less effectively detoxify and/or excrete xenobiotics than laboratory animals and thus are more vulnerable” (29). If toxicity is expected to be independent of basic metabolic rate (e.g., skin corrosion resulting from direct chemical reactivity), then allometric scaling is not appropriate.

#### Systemic effects

Allometric scaling factor recommendations are based on calculations accounting for differences in each species' body size in relation to humans. Suggested allometric scaling factors by ECETOC align with ECHA's recommendations (36) (for other species, consult Table R.8-3 in the ECHA guidance). While this approach is generally appropriate to account for interspecies differences, it should be modified if additional data on the substance or the species is known. It should be noted that this approach is appropriate for systemic toxicity following oral or dermal administration. It doesn't apply to direct local effects (i.e., skin or gastrointestinal irritation/corrosion), inhalation effects (local *or* systemic), or for doses in oral animal studies from the diet or in drinking water expressed as concentration in media (i.e., ppm in diet, mg/L in drinking water; dietary or drinking water exposures expressed in mg/kg/d would still apply allometric scaling). The rationale for the inhalation and oral dietary or drinking water concentration studies as exceptions to allometric scaling are justified in other guidance (29) (p. 23; for additional physiologically-driven restrictions on the use of allometric scaling, see p. 24–28).

For inhalation studies resulting in a systemic effect, no AF application is recommended where the principles of allometric scaling apply (note the limitations discussed in the above paragraph and in the PoD section) because breathing rates are anticipated to scale allometrically. However, owing to differences in experimental study design and occupational environments, it is appropriate to adjust for: (1) breathing rate differences between the test species (usually resting) and humans in the workplace (usually lightly respiring) and (2) number of days/hours the study includes compared to the average work week someone will experience in an occupational setting. These derivations are explained in full on page 8 of the ECETOC guidance (29), and are discussed in the PoD chapter of this guidance document.

REACH guidance suggests the use of an additional safety factor of 2.5 to account for any remaining interspecies differences in addition to the allometric scaling factor (36); ECETOC concludes that this additional variability is likely due to intraspecies differences that were inherent to the experimental design, and was therefore not recommended by ECETOC.

#### Local effects

Allometric scaling should not be applied since local effects (e.g., irritation) are not dependent upon metabolic rate (recommended interspecies AF of 1). For more information on the scientific basis and rationale to be considered for this type of effect, see ECETOC [(29), p. 28–29].

### Intraspecies

The intraspecies AF accounts for uncertainty resulting from differences in the underlying characteristics of the study population (e.g., age, gender, health status) compared to the diverse working population for whom the OEL is intended to protect. Size and composition of the study population are the two primary considerations when evaluating intraspecies uncertainty, with smaller, more homogenous studies requiring adjustment due to concerns that the average variability in the study population does not adequately represent the many unmeasured or unknown factors that affect human response in the target worker population.

For the purposes of OELs, ECETOC recommends an AF of 3 for worker populations (29), whereas ECHA recommends an AF of 5 (36), as an OEL is an exposure limit specifically pertaining to workplace exposures [for further explanation on this recommended difference, see Table 1 (29)]. This recommendation is held true for both systemic and local effects. The factor of 3 is expected to account for variability across a healthy population of working age, and is lower than the factor one would use if the effects observed in the key study were being applied to the general population (which inherently contains a higher degree of inter-individual variability). If there is reason to believe the working population would be uniquely susceptible to effects of exposure to the chemical/substance being evaluated, a higher AF may be considered and proposed, if substantiated with evidence.

For compounds studied using very large, diverse cohorts, or large meta-analyses, an assessment factor of 1 is considered appropriate. An AF of 1 may also be appropriate for study populations where sensitivity is well-defined and sensitive individuals are adequately represented in the study population. In addition, an intraspecies factor of 1–1.5 is generally a good starting point for intentional exposure studies of immediate, transient effects, such as irritation, which are usually associated with less inter-individual (i.e., intra-species) variation in response. However, because experimental studies are also relatively small (e.g., 10–20) and volunteers are usually younger and healthier than the average workplace population, the range of human variability may not be fully tested, necessitating a small intra-species AF.

### Exposure duration

The exposure duration AF accounts for extrapolation from a study design of shorter duration to a chronic exposure. This is important because an OEL needs to account for exposure across a number of years over a human's working lifespan, and the majority of animal studies occur within a much shorter time span. Because of this, an exposure duration AF is applied to account for any uncertainty in the extrapolation from a shorter term study in animals to longer term effects in humans. Essentially, the recommendation for the exposure duration AF is the same for both systemic and local effects. Scientific reasoning behind considerations for systemic and local effects, and why they are the same, can be found for the exposure duration AF in the ECETOC guidance (29). The table below details recommended ranges for default exposure duration study AFs (where subacute equates to a 28 day study, subchronic to a 90 day study, and chronic is a 1.5 year to lifetime study in a standard rodent assay):

There are instances where exposure duration AFs would need to account for not just the extrapolation of exposure duration based on study design, but additional aspects of the endpoint of interest itself as well. For example, expert judgement would need to be exercised in selecting the AF value if the NOAEL would decrease when an effect would be expected to become more severe with increasing exposure time, or if it would be expected that new effects would be likely if the study were extended out to a chronic exposure paradigm. For specific examples on what would drive these decisions and more information on where expert judgement should be applied, consult the ECETOC guidance document (29).

For human studies, the exposure AF generally accounts for uncertainty in one or more of the following: (1) insufficient exposure duration, (2) insufficient follow-up time, especially for long-latency endpoints such as most cancers, and/or (3) errors in exposure measurement/assessment and/or classification.

Uncertainty around insufficient exposure and/or follow up time are handled similarly. In the context of human data, ECETOC (26) recommended an AF of 2 where “sub/semi chronic effects are observed such as depression of blood counts or transitional chromosome aberrations following days/weeks of exposure, i.e., they are observable effects of possible pre-clinical significance and serve as a surrogate measure for frank effects”. However, to the extent possible, determination of an exposure AF should be data-driven. For example, if data exists that show that an exposure's effects increases by 40% after 20 years of exposure, due to an extremely long half-life, it could be reasonable to predict another 40% increase of this effect had exposure been extended out to 40 years, the maximum exposure time of a worker in the OEL setting. Thus, a data-derived AF of 1.4, which incorporates existing data, could be considered.

At least some degree of exposure measurement error and/or misclassification is present in virtually all epidemiologic studies and the uncertainty this source of error imparts into establishing a protective OEL derivation should be taken into consideration. Measurement error can occur as a result of either poor or inappropriate IH collection methods and procedures or limited retrospective exposure estimation. Measurement error can lead to exposure misclassification when individuals are assigned to categories of exposure (e.g., high, medium, low) that do not accurately reflect their true exposure level. Depending on how and when IH measurements were taken and how well those measurements correlate with actual individual level exposure (e.g., excursions, emergency response, maintenance), the direction of the error could lead to either an under- or over-estimation. If the health endpoint observed in the key study is attributed to an over-estimated exposure concentration, an AF greater than 1 is justified. Conversely, if effect estimates are associated with exposures that were under-estimated the AF should be <1. To the extent possible, a data-driven approach to identifying empirically derived AF are encouraged.

Where available, biomonitoring information can be helpful in assessing potential for under- and over-estimation of exposure from air measurements, especially in cases where respiratory protection was used (i.e., air monitoring data is not representative of the person's actual exposure) or where other routes of exposure are significant (e.g., dermal exposure which is often not quantitatively assessed). Biomonitoring may help reflect the total exposure, and in cases where correlations between biomonitoring values and air equivalent exposures are available, may be a more robust indicator of exposure depending on the specific chemical being considered.

### Dose-response (NOAEL-LOAEL extrapolation)

The dose-response AF takes into account potential differences in the dose response curve observed in the population under study to the dose response curve that is applicable to the target (working) population. Common complexities unique to the epidemiologic literature can complicate clear LOAEL/NOAEL identification and characterization of the dose-response curve, creating uncertainty around the selected PoD. In particular, continuous exposure data may preclude accurate identification of the concentration at which point risk increases above background. Wide and open-ended exposure categories may also limit clear identification of NOAEL/LOAEL. In addition, lack of monotonicity, whereby risk increases with each incremental dose or exposure category, creates further uncertainty about the robustness of observed associations.

For most well-designed toxicological studies, an AF of 3 will account for extrapolation from a LOAEL to a NOAEL [(29), Table 1; for further justification, see Table 1 (39); 7 studies are cited which detail ranges of the ratio of LOAEL/NOAEL for differing study durations and designs which substantiate the use of an AF of 3]. ECHA recommends a range of 1–10 for this AF (36). If the PoD from the key study is a NOAEL (or a BMDL, as this is considered equivalent to a NOAEL), an AF of 1 is suggested, as there are no adjustments to be made to account for uncertainties related to extrapolating from a LOAEL to a NOAEL. For further scientific evidence supporting the extrapolation AF value of 3 for LOAEL to NOAEL, refer to Section 2.2 of the ECETOC guidance (39). ECETOC states that the maximum value for LOAEL/NOAEL extrapolation generally is 10 but they considered that value as overly conservative; a larger AF should be considered where data indicate that a steep dose-response exists and/or for severe endpoints, thereby accounting for the greater consequence of any error in estimating the LOAEL/NOAEL (39).

Properties of the LOAEL or NOAEL that can influence justification to deviate from the recommended AFs to a higher value include: low study quality (note: different from quality of the whole database discussed in the next section), serious and/or irreversible effects, shallow dose-response curve (in which it's more difficult to determine where the true LOAEL/NOAEL lies), and dose-spacing higher than 2–4 fold (36). Consult the ECETOC 2010 guidance (29) for more detail on properties of the LOAEL or NOAEL that could justify deviation from these defaults, and whether these justifications apply specifically to the key study of interest.

### Quality of whole database

The database quality AF assignment includes a combination of a recommended range of acceptable values, and the expectation that expert judgement will be applied when selecting an appropriate AF for the key study.

When deciding whether to use the default AF of 1 for database quality, the following remaining uncertainties should be considered (39) in the potential assignment of a higher value than an AF of 1 (including, but not limited to): (1) completeness of the database, such that all endpoints potentially relevant to the compound of interest, both acute and/or chronic, have been adequately studied, (2) the use of a surrogate compound or compound(s), or the use of Quantitative Structure-Activity Relationship (QSAR)-derived information as a ‘read-across' to the substance being assessed, (3) consistency in the direction and magnitude of results across the body of data, (4) study quality (in the design, conduct, analysis, reporting) and (5) causal nature of the relationship, which would include but not be limited to: (a) potential deficiencies in the key study/studies such that confounders or effect modifiers were not adequately measured or analyzed, (b) whether appropriate statistical methods were used, (c) adequacy of sample size and study power, and (d) evaluation of bias, including the healthy worker/healthy worker survivor effect.

### Considerations for when to further refine AF based on available data

For substances with the type and/or specificity of toxicological data to deviate from default values recommended by ECHA and ECETOC guidance (29, 36, 39) (i.e., toxicokinetic and toxicodynamic data available), then chemical specific assessment factors (CSAFs) and more precise AFs may be considered. Some recommendations of ranges for AFs have been detailed above. For more information on what would specifically drive considerations for assignment of AFs based on considerable uncertainties, the respective sections of available guidance (29), and available literature, as AF application continues to be an ever-evolving space [for e.g., (5)].

## Discussion

Protecting worker health is a clear priority. Integrating information to meet this endeavor is a complex process which requires the combination of existing risk assessment frameworks and guidance as well as expert scientific judgment. Utilizing a multidisciplinary team of experts in epidemiology, toxicology, and exposure allows for a robust scientific process. This cross-disciplinary approach provides for the integration of substance-specific datasets (or read-across substances, when necessary) within the context of existing internationally recognized guidance and expert scientific judgment. The technical evaluation includes the following tenets of risk assessment: (1) problem formulation (2) literature review, (3) weight of evidence considerations, (4) point of departure, (PoD) selection/derivation, (5) application of assessment factors, and ultimately, the derivation of an OEL which is protective of worker health.

For more insight into how the OEL derivation framework could be applied in practice, consider the following example of chromium in specific conditions of use. The OEL derivation for Chromium (VI) [Cr(VI)] for welding and other “hot work” activities (e.g., torch-cutting, arc gouging) serves as a recent example of the applicability of the risk assessment principles detailed within this manuscript. There was a need identified to develop an OEL for Cr(VI) exposure to welders and those engaged in other “hot work” in ExxonMobil operations. While sodium dichromate is entirely hexavalent chromium, it was considered less relevant to the ExxonMobil occupational environment than chromium oxide exposure because it is a soluble form [unlike particulate chromium oxide dust (ACGIH 2017)], thus not expected to be representative of the form of chromium present from welding and thermal cutting/gouging processes. Problem formulation involved defining exposures relevant to welders as being within scope, which greatly limited the applicability of that dataset to the current question. Through the literature review and WoE process, it was determined that the form of Cr(VI) present in exposures during welding activities may be less toxic than during other types of occupational exposures (i.e., chromate production). Animal models exhibited quantitatively different responses as a function of different forms of hexavalent chromium (i.e., sodium dichromate vs. chromium oxide aerosols), and the studies offered limited precision in allowing for direct comparisons between the observed quantitatively different responses among different Cr(VI) forms.

There is sufficient information to support carcinogenic potential for hexavalent chromium in animal models. Observed tumor types appear largely restricted to the portal of entry. Drinking water exposures to sodium dichromate dihydrate resulted in clear evidence for carcinogenicity in both rats and mice (males and females affected similarly), with the tumor sites being the oral cavity (rats) or small intestine (mice). Due to the portal of entry dependence for carcinogenicity of chromium the OEL development focused on inhalation exposures to particulate, insoluble forms of chromium. The OEL recommendation for Cr(VI) is based on a chronic inhalation exposure of male Wistar rats (*n* = 18 exposed and *n* = 37 controls) to a 2:3 mixture of trivalent:hexavalent chromium oxide dust for 22–23 h/day, 7 days/week for 18 months, then monitored for up to 12 additional months (40). Chromium oxide dust was selected as the preferred form on the basis it is more likely to reflect chromium in fume generated from welding and thermal cutting/gouging processes. The measured concentration of Cr(VI) was reported to be 63.3 μg/m^3^ for the single group of rats exposed. No statistically significant effects on carcinogenic measures (number of rats with tumors, total tumor rate [benign or malignant]) were reported. Lung histopathology findings suggest 63.3 μg/m^3^ is a lowest observed adverse effect level (LOAEL) and served as the point of departure for OEL derivation. Applying assessment factors, the calculated value is 0.75 μg/m^3^, which was rounded to 1 μg/m^3^ per the SCOEL rounding guidance (41). Due to the limited nature of reported exposure levels of Cr(VI) and health outcomes among welder cohorts, the key study for this OEL derivation was based on animal data.

There is a need for transparency in the approach to OEL derivation, due to the amount and type of possible outcomes of the use of expert judgment. Utilizing existing risk assessment principles in a fit-for-purpose paradigm for OEL derivation is imperative in the pursuit of reproducibility of the process, especially in terms of the use of new and contemporary applications (e.g., integration of AOPs, literature search automation). These concepts were recently highlighted in a special issue on the state of the science of OEL development put forth in the Journal of Occupational and Environmental Health (42), which detailed contemporary advances in methodology and analysis of data relevant to OEL development, as well as a call for the use and implementation of advanced methods for OEL development. The approach to OEL derivation detailed in this manuscript are intended to integrate risk assessment principles tailored toward the needs of understanding how to utilize data to best protect worker health with state-of-the science approaches to those principles. OEL derivation techniques are evergreen processes which will evolve/modify over time as new operations, analyses/technologies and data emerge.

## Data availability statement

Publicly available datasets were analyzed in this study. This data can be found referenced in this manuscript: quantitative data and resources are listed as references in the reference list.

## Author contributions

LM and KG contributed to conception and design of the manuscript. LM, MA, AB, FG, RL, CN, NW, and KG each wrote sections of the manuscript. All authors contributed to manuscript revision, read, and approved the submitted version.

## Conflict of interest

The authors of this paper are or were employed by companies that manufacture petroleum products. The manuscript was written as part of normal employment and was the sole responsibility of the authors. No external funding was obtained for manuscript preparation.

## Publisher's note

All claims expressed in this article are solely those of the authors and do not necessarily represent those of their affiliated organizations, or those of the publisher, the editors and the reviewers. Any product that may be evaluated in this article, or claim that may be made by its manufacturer, is not guaranteed or endorsed by the publisher.

## Appendix

### Additional detail regarding confidence considerations for human health effects

For the LOE consisting of human health effects assessed in observational epidemiology studies, characteristics of studies that decrease confidence include:

- *Case reports*. These are often one-off situations resulting in catastrophic event such as knock-down or an unusual/severe clinical finding. Typically, neither the circumstance nor the level of exposure is relevant to OEL development. Additionally, the lack of a referent/control group represents a serious limitation with regards to inferences about exposure and the apical effects of interest.
- *Qualitative exposure metrics*. Ever/never, exposed/non-exposed, and high/medium/ low without some quantitative distinctions cannot inform an OEL. However, there might be situations in which the author provides a median/mean value for those categories. If those values are relatively close together, they might be useful in finding a NOAEL/LOAEL threshold (a POD). Values which, for example, vary by an order of magnitude are generally not helpful as that threshold/POD might exist anywhere along the broad within-category exposure continuum.
- *Inseparable components of mixtures*. Some types of chemical agents are ‘bundled together' when measuring workplace exposures despite significantly different levels of toxicity among sub-types that cannot be teased apart (e.g., benzene, toluene, ethylbenzene, and xylenes). Some authors make adjustments for this using various means, but those can be difficult to successfully execute.
- *Environmental studies (general population studies)*, typically, due to much lower concentrations and different exposure durations. These studies often lack individual-level data, so their utility is significantly limited.
- *Studies in which the study population represented has documented exposures to other stressors that are greater than those expected in the working population* (e.g., excessive smoking, alcohol/drug use). In this case, the study population may be more prone to show effects to the agent in question.

Characteristics of studies, often from the field of analytical epidemiology, that increase confidence include quantitative or measured exposures as a component of the overall exposure estimation, clearly described exposure history (e.g., exposure duration and age, cumulative exposure; average exposure; peak exposures, if available) including shape of the exposure distribution, air sampling that is representative of typical exposures (i.e, more than a single sample or a sample taken during documented IH excursions), short exposure category ranges to facilitate identification of NOAELs/LOAELs, accounts for co-exposures and other potential confounders in the workplace, sufficiently large study population sizes (e.g., large enough to result either in more than 5 expected cases for the key effect in the control/unexposed population or in confidence intervals that cover less than a two-fold range), and diverse study populations (e.g., multi-center trial or a meta-analysis of several studies from different geographical areas) (25).
